# A functional role of S100A4/non-muscle myosin IIA axis for pro-tumorigenic vascular functions in glioblastoma

**DOI:** 10.1186/s12964-022-00848-w

**Published:** 2022-04-07

**Authors:** Madoca Inukai, Ako Yokoi, Yuuki Ishizuka, Miki Hashimura, Toshihide Matsumoto, Yasuko Oguri, Mayu Nakagawa, Yu Ishibashi, Takashi Ito, Toshihiro Kumabe, Makoto Saegusa

**Affiliations:** 1 Department of Pathology, Kitasato University School of Medicine, 1-15-1 Kitasato, Minami-ku, Sagamihara, Kanagawa 252-0374 Japan; 2 Department of Neurosurgery, Kitasato University School of Medicine, 1-15-1 Kitasato, Minami-ku, Sagamihara, Kanagawa 252-0374 Japan; 3 Department of Pathology, Kitasato University School of Allied Health Science, 1-15-1 Kitasato, Minami-ku, Sagamihara, Kanagawa 252-0374 Japan

**Keywords:** Glioblastoma, S100A4, Non-muscle myosin IIA, Hypoxia, Recruitment, Cell migration

## Abstract

**Background:**

Glioblastoma (GBM) is the most aggressive form of brain tumor and has vascular-rich features. The S100A4/non-muscle myosin IIA (NMIIA) axis contributes to aggressive phenotypes in a variety of human malignancies, but little is known about its involvement in GBM tumorigenesis. Herein, we examined the role of the S100A4/NMIIA axis during tumor progression and vasculogenesis in GBM.

**Methods:**

We performed immunohistochemistry for S100A4, NMIIA, and two hypoxic markers, hypoxia-inducible factor-1α (HIF-1α) and carbonic anhydrase 9 (CA9), in samples from 94 GBM cases. The functional impact of S100A4 knockdown and hypoxia were also assessed using a GBM cell line.

**Results:**

In clinical GBM samples, overexpression of S100A4 and NMIIA was observed in both non-pseudopalisading (Ps) and Ps (-associated) perinecrotic lesions, consistent with stabilization of HIF-1α and CA9. CD34(+) microvascular densities (MVDs) and the interaction of S100A4 and NMIIA were significantly higher in non-Ps perinecrotic lesions compared to those in Ps perinecrotic areas. In non-Ps perinecrotic lesions, S100A4(+)/HIF-1α(−) GBM cells were recruited to the surface of preexisting host vessels in the vascular-rich areas. Elevated vascular endothelial growth factor A (VEGFA) mRNA expression was found in S100A4(+)/HIF-1α(+) GBM cells adjacent to the vascular-rich areas. In addition, GBM patients with high S100A4 protein expression had significantly worse OS and PFS than did patients with low S100A4 expression. Knockdown of S100A4 in the GBM cell line KS-1 decreased migration capability, concomitant with decreased Slug expression; the opposite effects were elicited by blebbistatin-dependent inhibition of NMIIA.

**Conclusion:**

S100A4(+)/HIF-1α(−) GBM cells are recruited to (and migrate along) preexisting vessels through inhibition of NMIIA activity. This is likely stimulated by extracellular VEGF that is released by S100A4(+)/HIF-1α(+) tumor cells in non-Ps perinecrotic lesions. In turn, these events engender tumor progression via acceleration of pro-tumorigenic vascular functions.

**Video abstract**

**Supplementary Information:**

The online version contains supplementary material available at 10.1186/s12964-022-00848-w.

## Background

Glioblastoma (GBM) is the most aggressive form of brain tumor with median overall survival of only 15.6 months with standard care (radiation and Temozolomide) and 20.5 months with additional treatment [1]. Tumor malignancy and clinical prognosis correlates with vascular-rich features, which are characterized by vascular proliferation in response to abundant vascular endothelial growth factor (VEGF) produced by tumor cells [2, 3]. The tumor vessels are morphologically and functionally different from normal blood vessels; they are tortuous, disorganized, and highly permeable, and have abnormalities in the endothelial walls, pericyte coverage, and basal cell membrane [4].

Glioma stem cells (GSC), constitute a subset of tumorigenic cancer stem cells (CSC), can self-renew and reconstitute a phenocopy of the original tumor; they are putative drivers of GBM malignancy and progression [5, 6]. In general, GSC reside within specialized tumor microenvironments or niches that maintain their stemness and malignant properties. Such environments include perivascular regions, hypoxic areas (adjacent to necrotic zones), and invasive niches [6–8]. GSC play an important role in tumor angiogenesis by inducing the formation of vascular endothelial cells and pericytes that may actively remodel perivascular niches [9, 10].

The S100 protein family is comprised of multigene calcium-binding proteins of the EF-hand type and has more than 25 members, each encoded by separate gene clusters on chromosome 1q21 [11, 12]. Although S100 family members are highly identical at the sequence and structural level, they are not functionally interchangeable [13–15]. S100A4 is frequently overexpressed in a variety of human malignancies and drives metastasis by stimulating angiogenesis, promoting the migration of tumor cells, and by facilitating the adhesion of cells to the extracellular matrix [16–19]. Although S100A4 possesses no enzymatic activity, it exerts its effects by interacting with and modulating the activity of other proteins, such as p53, non-muscle myosin IIA (NMIIA), and Annexin 2 to enhance tumor progression [20–22].

Here, we tested the hypothesis that the S100A4/NMMIA axis might contribute to GBM progression through modulation of tumor vascularization. We investigated the association between S100A4 and NMIIA expression, as well as the profiles of hypoxia-related molecules, and tumor vascularization status using clinical samples and cell lines of GBM.

## Methods

### Clinical cases

We selected 94 cases of primary isocitrate dehydrogenase (IDH) 1-wild type GBM that were surgically resected at Kitasato University Hospital between 2008 and 2020. Selection was based on the criteria of the 2016 World Health Organization classification of tumors of the central nervous system [23]. The mean age of the patients was 58.9 years (range 10–82 years). In GBM tissues, necrotic foci within tumor lesions were subdivided into two categories: necrosis with or without pseudopalisading (Ps) lesions, which are characterized by an accumulation of tumor cells around a central necrotic zone. Tumor lesions adjacent to non-Ps necrotic foci were also subdivided into three categories: adjacent, vascular-rich, and distal areas. None of the patients were treated with chemoradiation therapy before surgical resection of the tumors. All tissues were routinely fixed in 10% formalin and processed for embedding in paraffin wax. Approval for this study was given by the Ethics Committee of the Kitasato University School of Medicine (B20-197).

### Antibodies and reagents

Anti-hypoxia-inducible factor (HIF)-1α and anti-aldehyde dehydrogenase (ALDH) 1 antibodies were purchased from BD Biosciences (San Jose, CA, USA). Anti-CD34, anti-CD44s, anti-glial fibrillary acid protein (GFAP), and anti-human smooth muscle actin (SMA) antibodies were obtained from Dako (Copenhagen, Denmark). Anti-Snail and anti-Slug antibodies were from Cell Signaling Technology (Danvers, MA, USA). Anti-Sox2, anti-S100A4 (rabbit), anti-Twist 1, anti-Nestin, and anti-CD44v6 antibodies were obtained from Abcam (Cambridge, MA, USA). Anti-S100A4 (mouse), anti-carbonic anhydrase (CA) 9, and anti-NMIIA antibodies were from Proteintech (Rosemont, IL, USA). Anti-ZEB 1 and anti-β-actin antibodies were from Sigma-Aldrich Chemicals (St Louis, MA, USA). Blebbistatin and cobalt chloride (CoCl_2_) were purchased from Toronto Research Chemicals (North York, ON, Canada) and Sigma Chemical, respectively.

### Immunohistochemistry (IHC)

IHC was performed using a combination of the microwave-oven heating and polymer immunocomplex (Envision, Dako) methods as described previously [24–26].

For evaluation of IHC findings, scoring of nuclear or cytoplasmic immunoreactivities was performed as described previously [24–26]. Briefly, the proportion of immunopositive cells among the total number of counted cells was subdivided into five categories as follows: 0, all negative; 1, < 10%; 2, 10–30%; 3, 30–50%, and 4, > 50% positive cells. The immunointensity was also subclassified into four groups: 0, negative; 1, weak; 2, moderate, and 3, strong. IHC scores were generated by multiplication of the values of the two parameters.

Microvascular density (MVD) was examined as described by Hasan et al. [27], with minor modifications. Briefly, five high-power fields (HPFs) were randomly selected and CD34-immunopositive small vessels were counted in each area within tumor tissues. MVD was then calculated as the area of CD34-immunopsositivity per HPF. Based on the MVD data, tumor lesions adjacent to non-Ps necrotic foci were further subdivided into three categories: adjacent, vascular-rich, and distal areas. Briefly, vascular-rich lesions were defined as high MVD areas, whereas adjacent and distal areas were also subcategorized as lesions between massive necrosis and vascular-rich areas and lesions that were distal from vascular-rich areas within tumors, respectively.

### Immunofluorescence

The slides were heated in 10 mM citrate buffer (pH 6.0) for 3 × 5-min cycles using a microwave oven and then incubated overnight with anti-S100A4, anti-SMA, and anti-GFAP antibodies. Alexa 488, 570, and 647 (Thermo Fisher Scientific, Waltham, MA, USA) were used as secondary antibodies.

### In situ proximity ligation assay (PLA)

The slides were heated in 10 mM Tris–EDTA buffer (pH 9.0) for 3 × 5-min cycles using a microwave oven and then incubated overnight with anti-S100A4 (mouse) and anti-NMIIA (rabbit) antibodies. PLA was carried out the Duolink Detection kit with proximity ligation assay PLUS and MINUS probes for mouse and rabbit (Olink Bioscience, Uppsala, Sweden) as described previously [26]. The PLA signal score was determined on the basis of the percentage of PLA signal-positive cells and the PLA signal intensity with the multiplication of values of the two parameters as described previously [26].

### RNAscope assay for VEGFA mRNA in situ hybridization

VEGFA mRNA expression was analyzed using an RNAscope assay (Advanced Cell Diagnostics, Hayward, CA, USA) according to the manufacturer’s instructions. Briefly, deparaffined sections were incubated with the target retrieval for 15 min at 98 °C and were then treated by proteinase Plus for 30 min at 40 °C. The hybridization was performed with targeted probes: Hs-VEGFA (#423161), positive control probe (#2010684), and negative control probe (#310043) for 2 h at 40 °C. The signals were visualized with 3,3′ -diaminobenzidine and the nuclei were counterstained with methyl -green.

### Reverse transcription (RT)-PCR

cDNA was synthesized from 2 μg of total RNA. Amplification by RT-PCR was carried out in the exponential phase to allow comparison among cDNAs synthesized from identical reactions using specific primers. Primers for the *S100A4*, *VEGFA* and *GAPDH* genes were used as described previously [24–26].

### Plasmids and cell line

S100A4-specific shRNA, pGL3B-(− 1976/+ 1012) S100A4-luc, pGL3B-(− 2000/+ 50) VEGFA luc, and pcDNA3.1-HIF-1α were used as described previously [24–26, 28]. A promoter sequence of S100A4 (− 447/+ 1012), which was generated by a PCR strategy using a (− 1976/+ 1012) pGL3B-S100A4-luc construct, was cloned into the pGL-3 basic vector (Promega, Madison, WI, USA). Site-directed mutagenesis in the five putative HIF-1α binding sites in the (− 1976/+ 1012) or (− 447/+ 1012) S100A4-luc constructs was also performed using inverse PCR methods. The identity of all constructs was confirmed by sequencing prior to use. The sequences of PCR primers and mismatched oligonucleotides employed in this study are listed in the Table 1.

**Table 1:** Primer sequences of mutant *S100A4* promoter used in this study

Construct	Sequence	
Wild type 2	Forward	5′-TAGGCTGGTCTTGAACTCCTGGC-3′
(− 447/+ 1012)	Reverse	5′-GACAGCAGTCAGGATCTGGGAGCAGGAG-3′
Mut 1	Forward	5′-GGAGTATTCGCGCTCACTTGCCTGCCTCTGTCT-3′
	Reverse	5′-TGAGCGCGAATACTCCACAAAGTCCACCTGG-3′
Mut 2	Forward	5′-GCTGGGCCTTTCTCCCACACCCCCTCCTACCCT-3′
	Reverse	5′-GAGAAAGGCCCAGCCCCCGACAGCCAGCGC-3′
Mut 3	Forward	5′-TCTGGAAAGGCCCAGGTCTTCTGCGATCAGTTA-3′
	Reverse	5′-CCTGGGCCTTTCCAGATTGAAAGGGGTACAGGA-3′
Mut 4	Forward	5′-CACATGCGAATAAGACGGAGGAAAAAACAAACA-3′
	Reverse	5′-TCTTATTCGCATGTGTGTGTGTGCCATGCAC-3′
Mut 5	Forward	5′-ATAGTAAAGGTTGGTATGTATGTGCCTGTGGGT-3′
	Reverse	5′-ACCAACCTTTACTATAGCAACAGCGTGTGCAAG-3′

Underscored bases in primer sequences indicate mutations in the putative HIF-1α binding element

Mut, mutant type

GBM cell line KS-1 was obtained from the Health Science Research Resources Bank (Osaka, Japan) and was maintained in Eagle’s MEM with 10% bovine calf serum. S100A4-knockdown (KD) KS-1 cells (which have relatively high S100A4 expression) were established using an shRNA targeting the *S100A4* gene as described previously [25, 26]. For hypoxia experiments, cells were treated with 100 and/or 200 μM CoCl_2_ under 5% CO_2_ at 37 °C as described previously [29].

### Western blot assay and co-immunoprecipitation (IP)

Total cellular proteins were isolated using RIPA buffer [20 mM Tris–HCl (pH 7.2), 1% Nonidet P-40, 0.5% sodium deoxycholate, 0.1% sodium dodecyl sulfate]. Aliquots of the proteins were resolved by SDS-PAGE, transferred to membranes, and probed with primary antibodies that were then coupled with the ECL detection system (Amersham Pharmacia Biotech., Tokyo, Japan).

For co-IP, cells were lysed with IP buffer [10 mM Tris–HCl (pH 7.6), 100 mM NaCl, and 10% NP-40] in the presence of 1 mM CaCl_2_. Cell lysates were cleared and incubated with anti-S100A4 and NMIIA antibodies, followed by incubation with Protein G-Sepharose (Amersham Pharmacia Biotech). Western blot assay was subsequently performed with anti-S100A4 and anti-NMIIA antibodies.

### Aldefluor assay

ALDH 1 enzyme activity in viable cells was determined using a fluorogenic dye-based Aldefluor assay (Stem Cell Technologies, Grenoble, France), according to the manufacturer’s instructions. The prepared cells were analyzed by flow cytometry using BD FACS Calibur (BD Biosciences) and CellQuest Pro software (BD Biosciences).

### Spheroid assay

Cells (1 × 10^3^) were plated in low cell binding plates (Thermo Fisher Scientific, Yokohama, Japan) in Neuralbasal medium (Thermo Fisher Scientific, Waltham, MA, USA). Uniform spheroids with a minimum diameter of 50 μm were counted approximately 2 weeks following plating.

### Wound healing assay

Cells were seeded into 24-well tissue culture plates and grown to reach almost total confluence. After a cell monolayer formed, a scratch wound was made with a sterile 200-μl tip. The area of the wound was analyzed with ImageJ software version 1.41 (NIH). Cell migration was calculated based on the number of pixels occupied by the wound closure compared to control scratches.

### Migration assay

Cell migration was determined using 24-well transwell chambers with 8-μm pore size (Corning, NY, USA). The lower chamber was filled with medium containing 10% serum. Cells were suspended in the serum-free upper medium and transferred into the upper chamber. After 48 h, the number of HE-stained cells on the bottom surface of the polycarbonate membranes was counted using a light microscope.

### TCGA data analysis

mRNA expression data (RNA Seq V2 PSEM) for the *S100A4* and *myosin heavy chain (MYH) 9* (also known as NMIIA) genes in 144 GBM cases were extracted from cBioportal for Cancer Genomics (http://www.cbioportal.org/). The data were subcategorized into ‘high’ and ‘low’ groups (scores > 0 and 0, respectively) based on the median Z score (= 0) for mRNA expression levels in each category, and then examined for any correlation with overall survival (OS) or progression-free survival (PFS).

### Statistics

Comparative data were analyzed using the Mann–Whitney *U*-test and the Spearman’s correlation coefficient. Overall survival (OS) was calculated as the time between onset and death of the date or the last follow-up evaluation. Progression-free survival (PFS) was also examined from the onset of treatment until relapse, disease progression, or last follow-up evaluation. OS and PFS were estimated using the Kaplan–Meier method, and statistical comparisons were made using the log-rank test. Univariate analysis was also performed using the Cox proportion hazards regression model. The cutoff for statistical significance was set as *P* < 0.05.

## Results

### Overexpression of S100A4 in response to hypoxic effects in GBMs

**Fig. 1:**
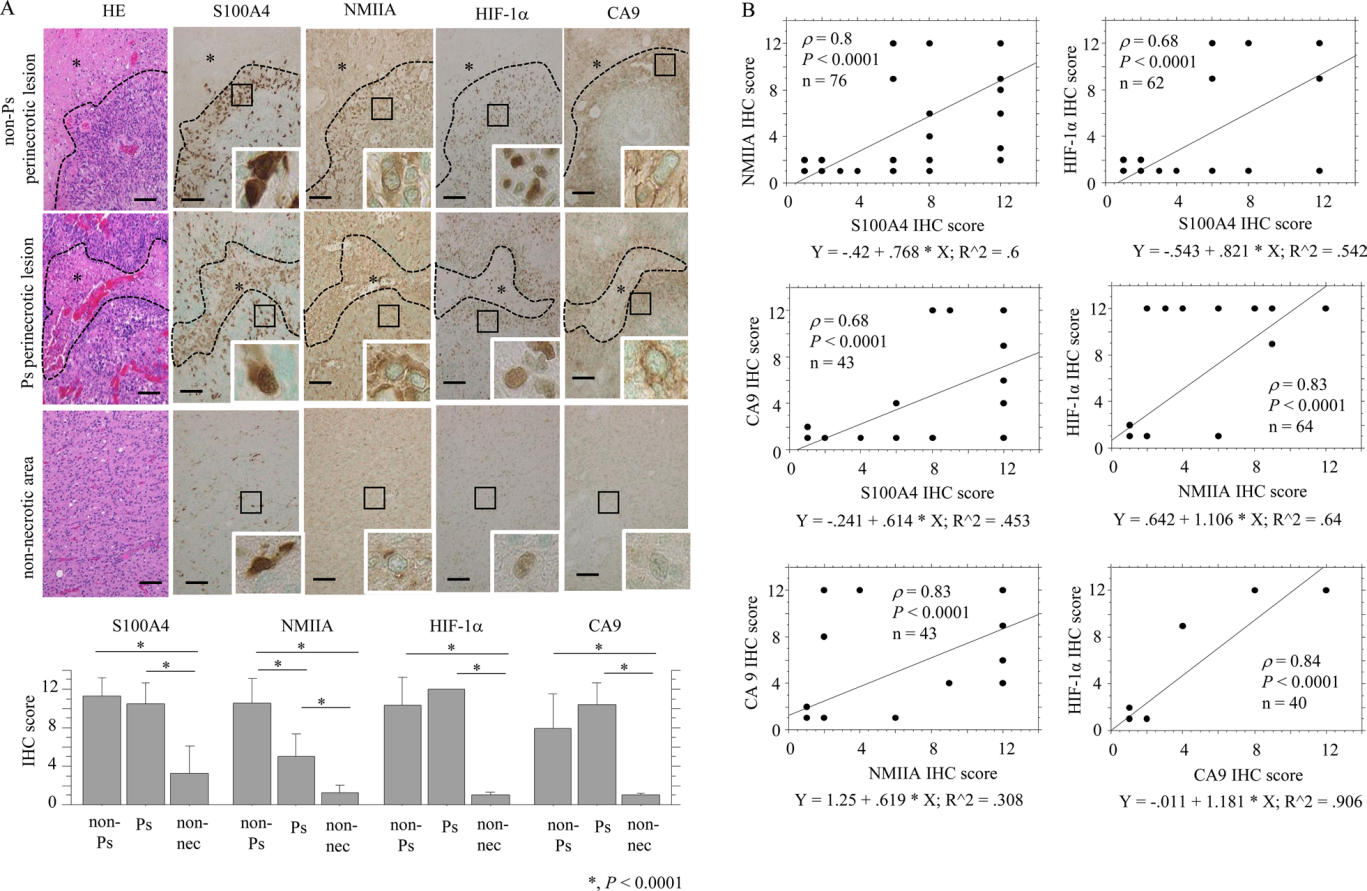
Overexpression of S100A4 under hypoxic conditions in GBMs. **A** Upper: staining by HE and IHC for the indicated proteins in GBMs. Note the strong immunoreactivity for S100A4, NMIIA, HIF-1α, and CA9 in both non-Ps and Ps perinecrotic lesions. Necrotic areas are indicated by asterisks and partitioned by dotted lines. Insets show the magnified views of the boxed area. Original magnification, × 100 and × 400 (inset). Scale bar = 200 μm. Lower: IHC score for the indicated molecules in non-Ps and Ps perinecrotic (non-Ps and Ps) and non-necrotic lesions (non-nec). The data are presented as means ± SDs. **B** Correlation between IHC scores of S100A4 and related molecules in GBM. *ρ*, Spearman’s correlation coefficient; n, number of cases

Representative images of IHC findings for S100A4, NMIIA, HIF-1α, and CA9 in GBMs are illustrated in Fig. 1A. Nuclear/cytoplasmic immunoreactivity for S100A4, nuclear immunoreactivity for HIF-1α, and cytoplasmic immunostaining for NMIIA and CA9 were frequently found in GBM cells that were mainly located at both non-Ps and Ps (-associated) perinecrotic lesions. Average IHC scores for these markers were significantly higher in non-Ps perinecrotic and Ps lesions when compared to those of non-necrotic tumor areas (Fig. 1A). In addition, S100A4 score was positively correlated with NMIIA, HIF-1α, and CA9 scores. NMIIA score was also positively correlated with HIF-1α and CA9 scores (Fig. 1B).

**Fig. 2:**
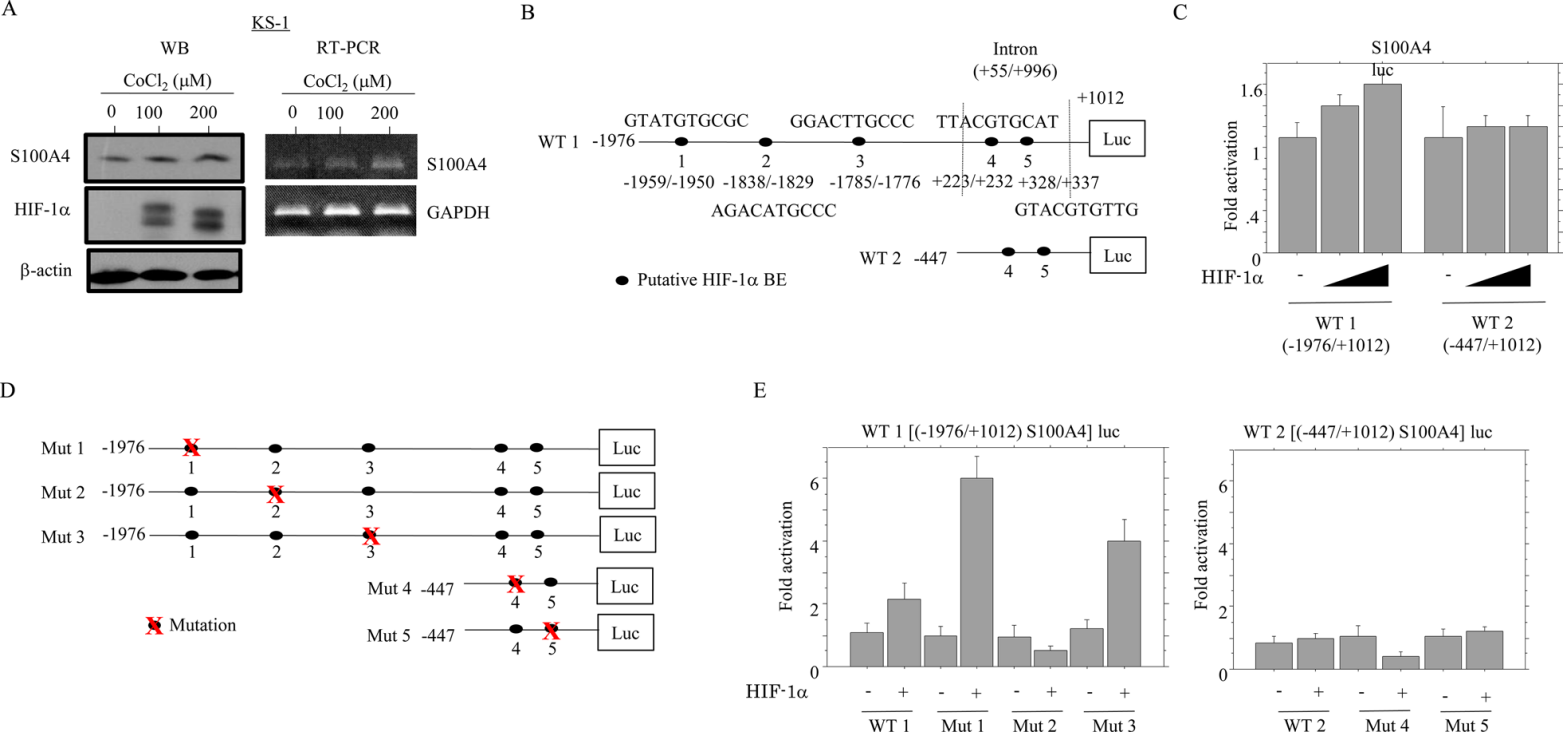
Transcriptional upregulation of S100A4 by HIF-1α. **A** Western blot (left) and RT-PCR analyses (right) for the indicated molecules in total lysates or total RNA from KS-1 cells treated with 100 and 200 μM CoCl_2_ for 24 h. **B** The *S100A4* promoter sequence containing five putative HIF-1α -binding sites (BEs). **C** KS-1 cells were transfected with Wild type (WT) 1 (− 1986/+ 1012) and WT 2 (− 447/+ 1012) S100A4-luc reporter constructs, together with HIF-1α. Relative activity was determined based on arbitrary luciferase light units normalized to pRL-TK activity. The activities of the reporter plus the effector relative to that of the reporter plus empty vector are shown as means ± SDs. The experiment was performed in duplicate. **D** Various promoter constructs with mutations (Mut, mutant-type) in the putative HIF-1α-BEs were used for evaluating transcriptional regulation of the *S100A4* promoter by HIF-1α. **E** KS-1 cells were transfected with various mutant constructs of S100A4 promoter, along with HIF-1α

In KS-1 cells, treatment with the hypoxia mimetic CoCl_2_ increased the expression of S100A4 and HIF-1α (Fig. 2A); consistent with this, the expression of S100A4 mRNA was increased. A search of the *S100A4* promoter for potential HIF-1α binding sites (a consensus core motif of 5′-A/GCGTG-3′) revealed the presence of five sites, at − 1959 to − 1950 (site 1), − 1838 to − 1829 (site 2), − 1785 to − 1776 (site 3), and + 223 to + 232 (site 4) (Fig. 2B), as well as + 328 to + 337 (site 5) in the first intron as previously reported [30]. The Wild type (WT) 1 (− 1976/+ 1012) *S100A4* promoter construct was activated ~ 1.6 fold following transfection of HIF-1α, whereas such an effect was not evident in WT 2 (− 447/+ 1012) *S100A4* promoter (Fig. 2C). Moreover, the *S100A4* promoter was significantly less responsive to HIF-1α when a 3-nucleotide alteration was introduced into site 2, but not sites 1, 3, 4, and 5 (Fig. 2D,E). Together, these data suggest that the region from − 1838 to − 1829 may constitute a functional *cis*-acting element necessary and sufficient for *S100A4* promoter stimulation by HIF-1α.

These findings suggest that increased S100A4 expression, which is at least partly driven by HIF-1α-transactivation, is found in perinecrotic lesions of GBM tissues under hypoxic conditions.

### S100A4 is associated with cell migration through an interaction with NMIIA

**Fig. 3:**
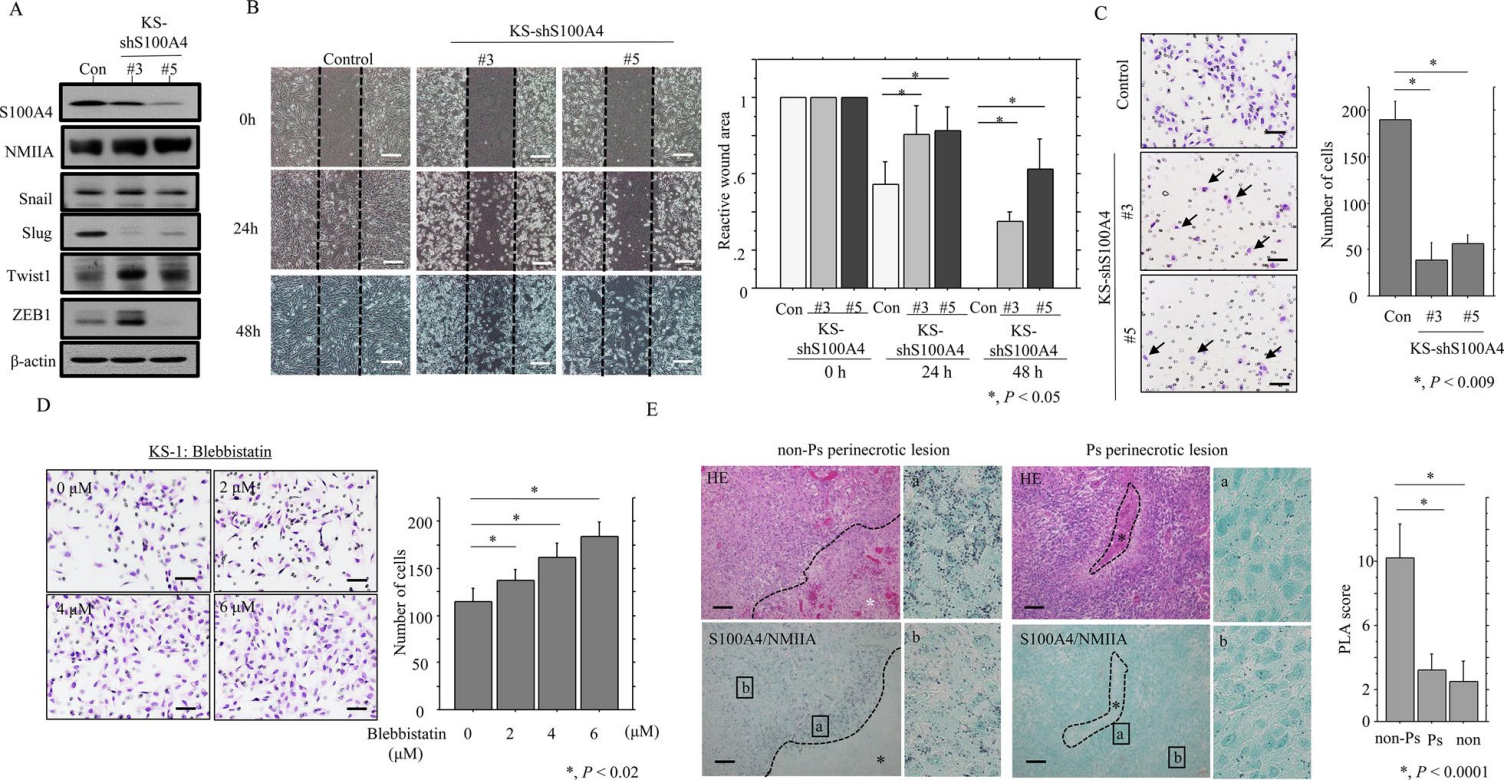
Changes in cell migration ability following knockdown of S100A4 and inhibition of NMIIA activity in GBM cells. **A** Western blot analysis for the indicated proteins of total lysates from S100A4-knockdown KS-1 (KS-shS100A4#3 and #5) and control cells (Con). **B** Left: wound-healing assay with S100A4-knockdown KS-1 and control cells (Con). A scratch was made in the middle of confluently grown cells, and phase -contrast images were taken after 0 h (upper), 24 h (middle) and 48 h (lower). Scale bar = 100 μm. Right: the wound areas were calculated using Image J software version 1.41, with the area at 0 h post-wounding set as 1. The fold wound areas are presented as means ± SDs. **C** Left: migration rate measured using a transwell assay. The S100A4-knockdown KS-1 and control cells (Con) were seeded in a 24-well transwell plate and incubated for 24 h in medium without serum. Cells were stained by HE and counted using a light microscope. Scale bar = 100 μm. Right: cell numbers are presented as means ± SDs (lower). **D** Left: KS-1 cells were seeded in a 24-well transwell plate and incubated for 24 h in serum-free medium with 0, 2, 4, and 6 μM blebbistatin. Cells were stained by HE and counted using a light microscope. Scale bar = 100 μm. Right: cell numbers are presented as means ± SDs. **E** Left and middle: in situ PLA assay for the S100A4-NMIIA interaction in non-Ps and Ps perinecrotic lesions of GBMs. Note the small aggregated dots in cytoplasmic and nuclear compartments of the GBM cells in non-Ps, but not Ps, perinecrotic lesions. Necrotic areas are indicated by asterisks and partitioned by dotted lines. Insets show the magnified views of the boxed area. Original magnification, × 100 and × 400 (inset). Scale bar = 200 μm. Right: PLA score for the combinations of S100A4 and NMIIA in non-Ps and Ps perinecrotic (non-Ps and Ps) and non-necrotic lesions (non). The data are presented as means ± SDs

To examine whether S100A4 contributes to GBM cell migration, we established two independent KS-1 cell line clones in which S100A4 expression was blocked by S100A4-specific shRNAs (KS-shS100A4#3 and #5). Expression of Slug, but not Snail, Twist1, or ZEB1, as well as NMIIA, was reduced in KS-S100A4-knockdown (KD) cells when compared to control cells (Fig. 3A). The KS-S100A4-KD cells refilled wounded empty spaces more slowly (Fig. 3B), in line with their significantly decreased migration rates as compared with control cells (Fig. 3C).

Because the biological activity of S100A4 is based on interactions with potential binding partners [20], we evaluated the function of NMIIA, a major partner for S100A4 [20], in KS-1 cells. To this end, we used blebbistatin, a synthetic chemical compound that effectively and reversibly blocks the ATPase activity of NMII without inhibiting class I, V, and X myosin superfamilies [31]. Treatment with the compound significantly enhanced cellular migration capacity in a dose-dependent manner (Fig. 3D).

To further examine the interaction between S100A4 and NMIIA, we performed an in situ PLA assay using antibodies targeting S100A4 and NMIIA in 10 GBM cases. Interactions of the two molecules were predominantly observed in the cytoplasmic compartments of GBM cells. The PLA score was significantly higher in non-Ps perinecrotic lesions when compared to those of Ps lesions and non-necrotic tumor areas (Fig. 3E). This association was confirmed by co-IP of S100A4 and NMIIA in KS-1 cells (Additional file 1: Figure S1).

Since S100A4 is a normal stemness marker [32, 33], we also examined whether there were an association between S100A4 and GSC properties. Immunoreactivity for Nestin, which is a neuronal stem cell marker [3], was significantly higher in non-Ps and Ps perinecrotic lesions as compared to non-necrotic areas in GBM tissues (Additional file 2: Figure S2A). Nestin expression was also positively correlated with S100A4, NMIIA, HIF-1α, and CA9 scores (Additional file 2: Figure S2B). Treatment of KS-1 cells with CoCl_2_ increased the expression of Nestin and HIF-1α (Additional file 3: S3A). However, S100A4-KD did not alter the expression of several CSC-related markers, spheroid formation, or ALDH1^high^ population (Additional file 3: Figure S3B,C,D).

These findings suggest that S100A4 enhances cell mobility via inhibition of NMIIA, but does not regulate GSC properties in KS-1 cells.

### S100A4 is associated with vascularization in non-Ps perinecrotic lesions of GBM

**Fig. 4:**
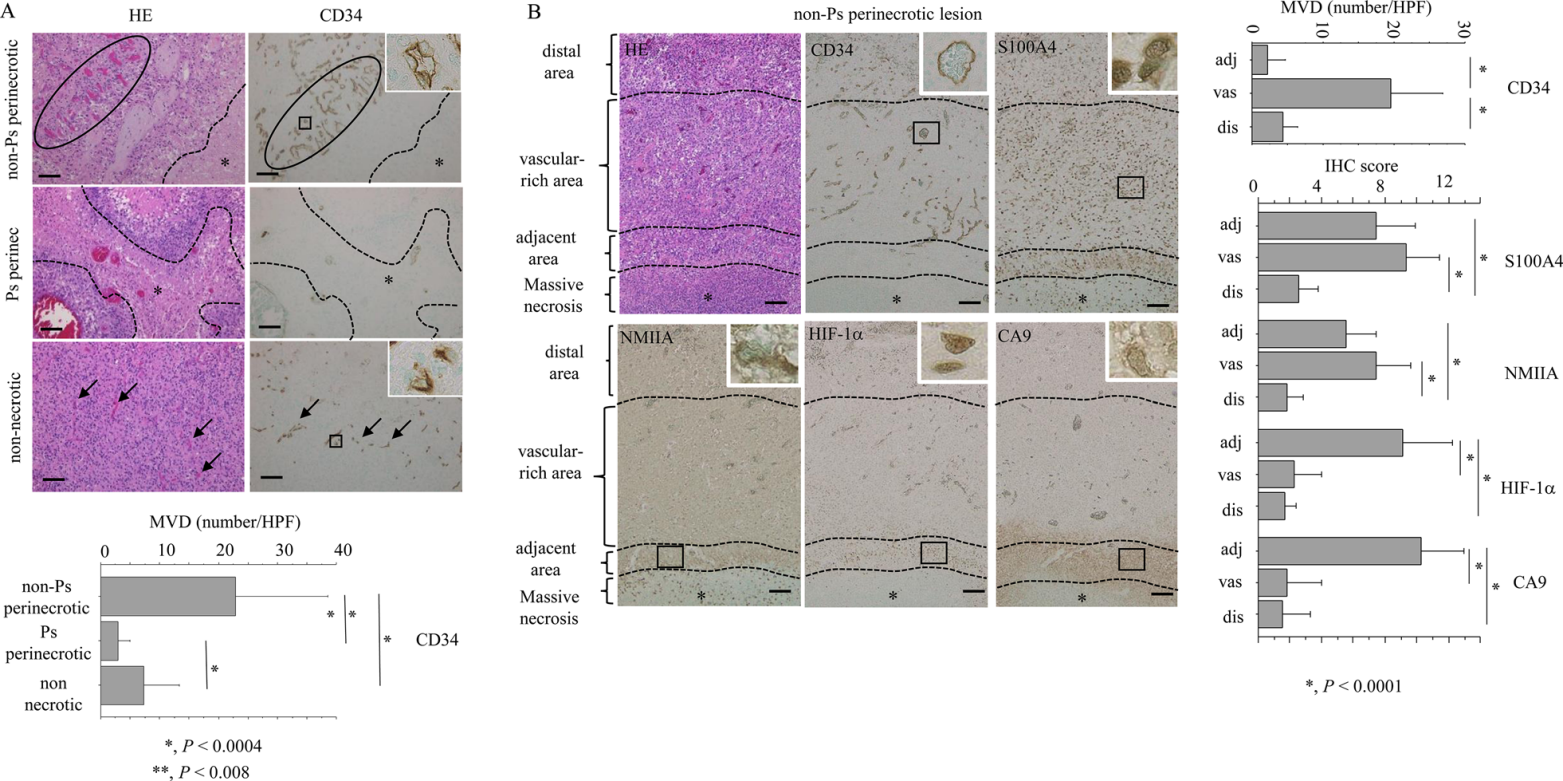
Association between S100A4 expression and vascularization in GBM. **A** Upper: HE staining and IHC for CD34 in non-Ps and Ps perinecrotic and non-necrotic lesions of GBMs. Note the dense CD34 immunoreactivity (closed circle) in non-Ps perinecrotic lesions, in contrast to the scattered immunopositivity (indicated by arrows) in Ps perinecrotic and non-necrotic lesions. Necrotic areas are indicated by asterisks and partitioned by dotted lines. Original magnification, × 100. Scale bar = 200 μm. Lower: microvascular density (MVD) determined by CD34 immunoreactivity in non-Ps and Ps perinecrotic and non-necrotic lesions. The data are presented as means ± SDs. **B** Left: staining by HE and IHC for the indicate molecules in adjacent, vascular-rich, and distal areas of non-Ps perinecrotic lesions of GBMs. The three lesions are partitioned by dotted lines. Necrotic areas are indicated by asterisks. Insets show the magnified views of the boxed area. Original magnification, × 100 and × 400 (inset). Scale bar = 200 μm. Right: microvascular density (MVD) determined by CD34 immunoreactivity (upper) and IHC score for the indicated molecules in adjacent (adj), vascular-rich (vas), and distal (dis) areas of non-Ps perinecrotic lesions of GBMs. The data are presented as means ± SDs

Since activation of HIF-1 signaling contributes to the intense vascular hyperplasia often seen in GBM [34], we first examined vascular status in both non-Ps and Ps perinecrotic lesions. CD34(+) small vessels were frequently observed in non-Ps perinecrotic lesions when compared to those of Ps perinecrotic and non-necrotic lesions (Fig. 4A). The differences in microvascular densities (MVDs) as determined by CD34 immunohistochemistry were statistically significant (Fig. 4A).

Based on the above findings, we subdivided non-Ps perinecrotic lesions into three categories: those adjacent to massive necrotic areas within tumors, vascular-rich lesions, and distal lesions (Fig. 4B). MVDs were significantly higher in vascular-rich areas when compared to those of adjacent and distal lesions. S100A4 and NMIIA scores were significantly higher in both adjacent and vascular-rich areas compared to distal lesions, whereas HIF-1α and CA9 scores were significantly higher in adjacent lesions than in vascular-rich and distant lesions (Fig. 4B).

**Fig. 5:**
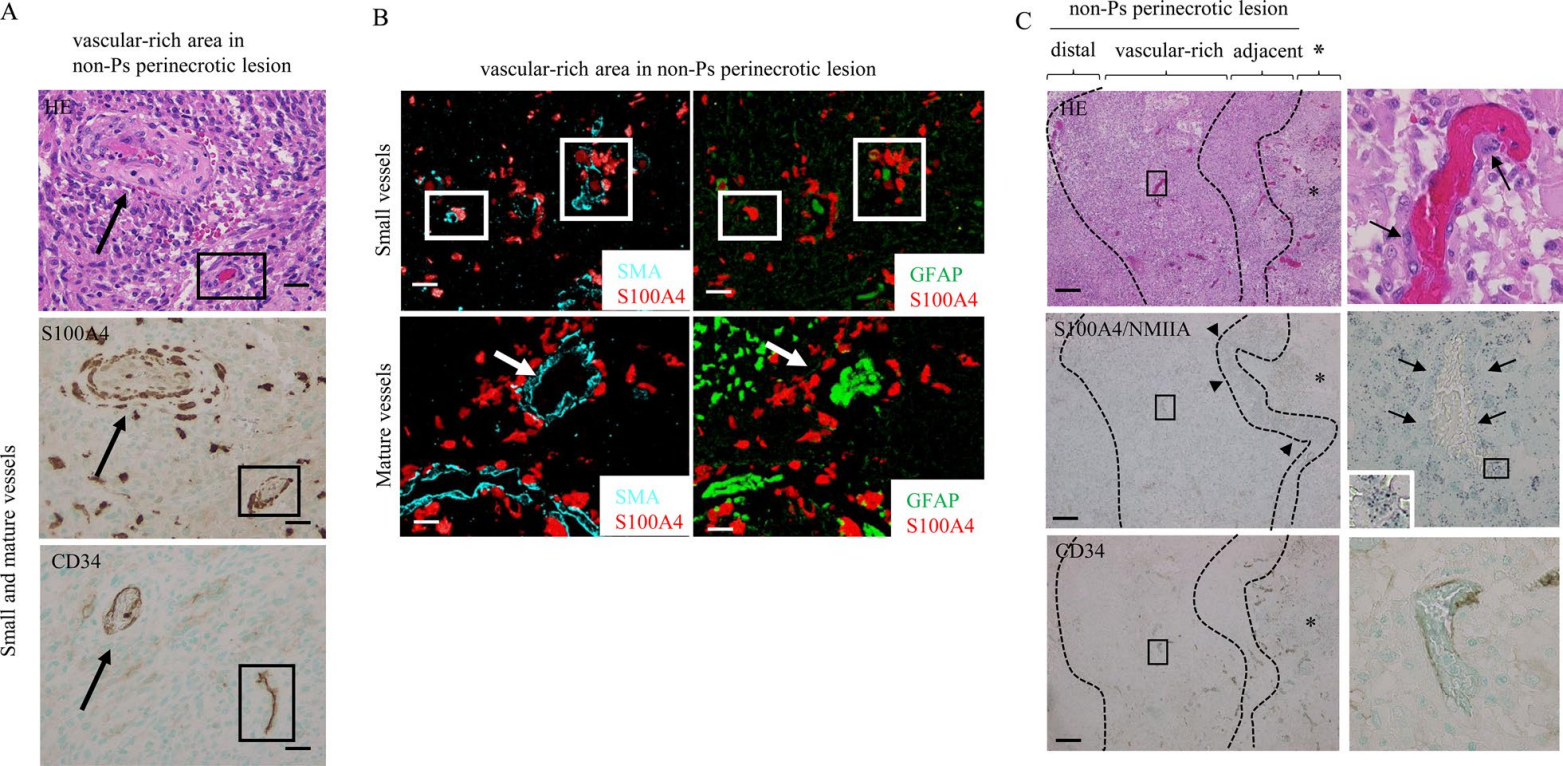
Vascular co-option by S100A4-positive GBM cells and VEGFA mRNA expression in HIF-1α-positive tumor cells. **A** Staining by HE (upper) and IHC for the indicated proteins (middle and lower) in vascular-rich areas of non-Ps perinecrotic lesions in GBMs. S100A4-positive GBM cells that are co-opted along the surface of preexisting CD34-positive small (indicated by boxes) and mature vessels (indicated by arrows). Original magnification, × 200. Scale bar = 100 μm. **B** Immunofluorescence for the indicated proteins in vascular-rich area in non-Ps perinecrotic lesion. S100A4/GFAP-positive GBM cells around both small (middle panels: indicated by boxes) and mature vessels (lower panels: indicated by arrows) along SMA-positive vascular components. Original magnification, × 200. Scale bar = 100 μm. **C** Staining by HE (upper), in situ PLA assay for the S100A4-NMIIA interaction (middle), and IHC for CD34 (lower) in vascular-rich area of a non-Ps perinecrotic lesion in GBM using semiserial sections. Note the small aggregated dots in GBM cells around small vessels (indicated by arrows), as well as those in adjacent areas (indicated by arrow-heads). The three lesions including adjacent, vascular-rich, and distal areas are partitioned by dotted lines. Necrotic areas are indicated by asterisks. Right panels show the magnified views of the boxed areas in the left panels. Original magnification, × 100 (left panels) and × 400 (right panels). Scale bar = 200 μm

**Fig. 6:**
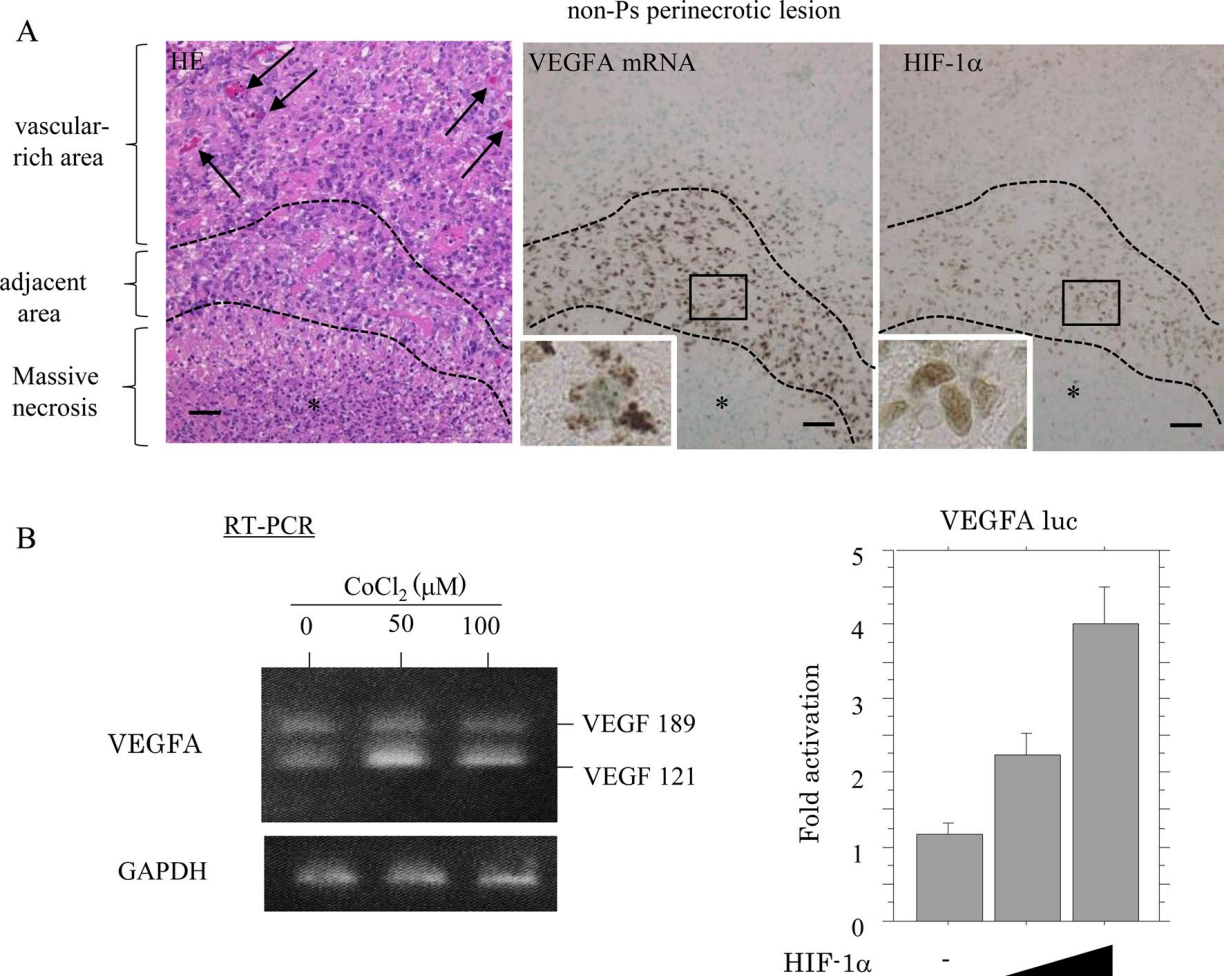
Overexpression of VEGFA by HIF-1α in GBM cells. **A** Staining by HE (left), RNAscope for VEGFA mRNA (middle), and IHC for HIF-1α (right) in non-Ps perinecrotic lesions of GBM. Note the dense vascular structures (indicated by arrows) in the vascular-rich area (left) and the high VEGFA mRNA and HIF-1α immunopositivity in GBM cells in the adjacent area (partitioned by dotted lines) (middle and right). Necrotic areas are indicated by asterisks and partitioned by dotted lines. Insets show the magnified views of the boxed area. Original magnification, × 100 and × 400 (inset). Scale bar = 200 μm. **B** Left: RT-PCR analysis of VEGFA mRNA levels in KS-1 cells after 50 and 100 μM CoCl_2_ treatment for 24 h. Right: KS-1 cells were transfected with VEGFA-luc reporter constructs, together with 100 and 250 ng HIF-1α. Relative activity was determined based on arbitrary luciferase light units normalized to pRL-TK activity. The activities of the reporter plus the effector relative to that of the reporter plus empty vector are shown as means ± SDs. The experiment was performed in duplicate

Finally, strong S100A4(+)/GFAP(+) GBM cells showed vessel co-option, where tumor cells migrate along the preexisting vessel [35], around both small- and mature vascular structures with strong SMA(+) vascular components in the vascular-rich area (Fig. 5A,B). An in situ PLA assay also revealed the interaction of S100A4 and NMIIA in GBM cells that are co-opted along the surface of vascular components (Fig. 5C). In contrast, tumor cells expressing VEGFA mRNA, along with nuclear HIF-1α, were found in adjacent, but not vascular, lesions (Fig. 6A). In KS-1 cells, treatment with CoCl_2_ increased VEGFA mRNA expression, consistent with activation of the *VEGFA* promoter following cotransfection of HIF-1α (Fig. 6B).

These findings suggest that S100A4(+)/HIF-1α(−) GBM cells are co-opted along preexisting vessels in the vascular-rich area; they are adjacent to S100A4(+)/HIF-1α(+) tumor cells with activated HIF-1α/VEGF signaling in non-Ps perinecrotic lesions.

### S100A4 is associated with unfavorable prognosis in GBM

**Fig. 7:**
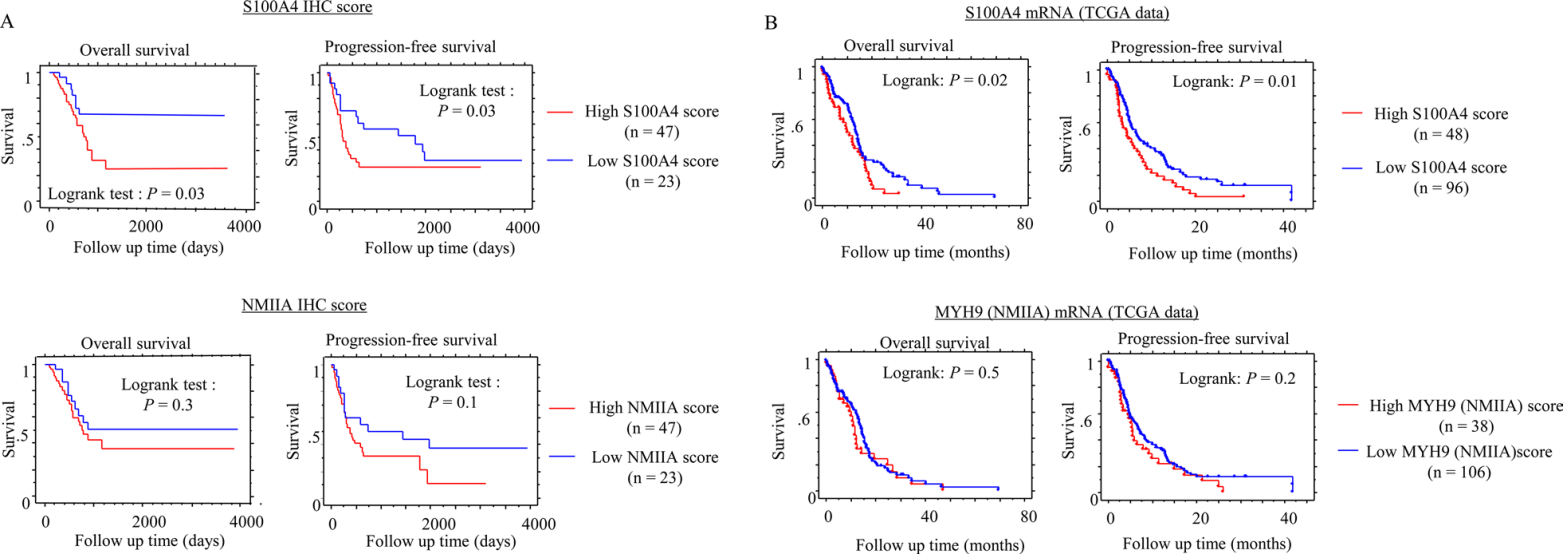
Relationship between S100A4 and NMIIA expression and prognosis in GBMs. **A** OS (left) and PFS (right) as a function of S100A4 and NMIIA protein expression (upper and lower) in GBM. **B** TCGA data analyses for OS (left) and PFS (right) as a function of S100A4 and NMIIA mRNA expression (upper and lower) in the GBMs

To evaluate the prognostic significance of S100A4 and its related markers, the IHC scores were divided into two categories (high and low) based on the cut-off values (mean scores) (Table 2). Kaplan–Meier analysis revealed that, in GBM cases, patients with high S100A4 scores had poorer OS and PFS when compared to patients with low scores, but such associations were not observed with regard to NMIIA scores (Fig. 7A). We made similar observations when we analyzed the TCGA data for expression of S100A4 and MYH9 (NMIIA) mRNA (Fig. 7B). Of five IHC markers and two clinical factors, univariate Cox progression hazards regression revealed that only S100A4 score was a significant prognostic factor for OS and PFS in GBM (Table 2).

**Table 2:** Univariate analyses for overall survival and progression-free survival in GBM

Variable	Univariate analysis			
	Cut-off	Log rank c2	*P*-value	Unfavorable factor
*Overall survival*				
S100A4	7/8	16.6	< 0.0001	High score
NM IIA	5/6	1.7	0.2	
Nestin	7/8	0.02	0.9	
CA9	7/8	1.9	0.2	
HIF-1α	7/8	0.6	0.4	
Gender	M/F	1.1	0.3	
Age	58/59	0.06	0.8	
*Progression-free survival*				
S100A4	7/8	5.1	0.02	High score
NM IIA	5/6	2.6	0.1	
Nestin	7/8	0.008	0.9	
CA9	7/8	0.03	0.8	
HIF-1α	7/8	0.02	0.9	
Gender	M/F	0.3	0.6	
Age	58/59	1.7	0.2	

IHC scores are divided into two categories (high and low) based on the cut-off values (mean scores)

## Discussion

The present study clearly provides evidence that overexpression of S100A4, as well as NMIIA, occurs in both non-PS and Ps perinecrotic lesions and is positively correlated with HIF-1α and CA9 expression in GBM; these findings are consistent with those of other studies in esophageal and gastric carcinomas [30, 36]. Inducing hypoxia in KS-1 cells led to the upregulation of S100A4 at both the mRNA and protein level, in line with stabilization of HIF-1α protein. In addition, we demonstrated that HIF-1α can specifically activate transcription of an *S100A4* promoter through the HIF-1α binding site, probably located at − 1838 to − 1829; this is in contrast to earlier study that used a gastric carcinoma cell line [30]. One possible reason for the discrepant results may be that transactivation of *S100A4* requires cell type-specific factors.

Interestingly, S100A4(+)/HIF-1α(+) GBM cells were found near non-Ps perinecrotic lesions, whereas S100A4(+)/HIF-1α(−) tumor cells were located at the adjacent vascular-rich areas. Since *S100A4* is transcriptionally upregulated through hypoxia-induced activation of NF-κB signaling [25, 37, 38], it appears that the regulation of S100A4 expression in hypoxic regions of GBM is multifactorial. In fact, STAT3 works with HIF1 to activate HIF1 target genes and to drive HIF-1-dependent tumorigenesis under hypoxic conditions [39].

We also found that S100A4 knockdown was closely associated with decreased migration capability, along with reduced Slug expression, which is a major indicator for epithelial-mesenchymal transition [40]. The opposite effects were elicited following blebbistatin-dependent inhibition of NMIIA activity. An interaction between S100A4 and NMIIA was observed in lysates from KS-1 cells, and by using in situ PLA we confirmed that this interaction was mainly in the cytoplasm of tumor cells, particularly in non-Ps perinecrotic lesions. Because S100A4 can abrogate the actin-dependent ATPase and polymerization activity of NMIIA [41, 42], S100A4 may serve as a critical regulator of tumor migration, in line with the idea that NMIIA acts as an inhibitor of cell migration [43]. Furthermore, the PLA score was significantly higher in non-Ps perinecrotic lesions, which suggests these lesions may harbor other microenvironmental factors associated with tumor migration ability. Further studies will be required in order to elucidate these factors.

Importantly, S100A4(+)/HIF-1α(−) tumor cells were recruited along the surface of host preexisting vessels in vascular-rich areas of non-Ps perinecrotic lesions, whereas high VEGFA mRNA expression was found in S100A4(+)/HIF-1α(+) GBM cells in the adjacent areas. Although co-opting host vessels is just one strategy that tumors can employ to survive, it can be essential in some instances [44]. The expression of recombinant VEGF-A_165_ alters the architecture and function of the co-opted preexisting brain vasculature in melanoma brain metastases [45]. Similarly, therefore, we suggest that S100A4-driven alteration of preexisting vasculature may play a critical role in GBM progression, perhaps by accessing an alternative tumor blood supply in the presence of VEGFA. This hypothesis is supported by data herein showing that high S100A4 expression was associated with significantly worse OS and PFS than low S100A4 expression, and by the results of our TGCA data analysis.

Finally, high Nestin score was significantly associated with hypoxia in GBM tissues, as well as in the KS-1 cell line, which is consistent with reports of an increased CSC fraction and acquisition of a stem-like state following oxygen deprivation [5, 6]. Since S100A4 and Nestin scores are positively correlated in GBM and S100A4 is reportedly a critical regulator of the epithelial to mesenchymal transition in GSC [46], we predicted that S100A4 may be a critical regulator of GSC. However, S100A4 knockdown did not elicit changes in several GSC properties. We therefore suggest that the role of S100A4 in modulation of GSC features is only manifest when S100A4 is overexpressed.

## Conclusion

**Fig. 8:**
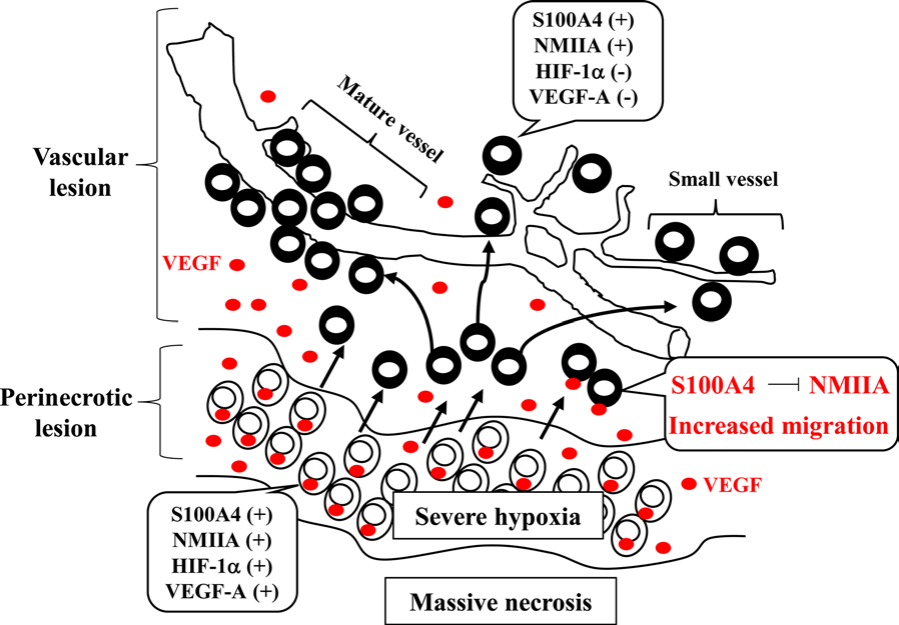
Schematic representation of the role of the S100A4/NMIIA axis in GBM. Aggressive and poor prognostic features are elicited through enhancement of tumor cell mobility and acceleration of vascularization

Our results suggest a novel functional role of the S100A4/NMIIA axis in GBM (Fig. 8). Following severe hypoxia, S100A4 is upregulated and interacts with NMIIA; this inhibits NMIIA activity and thus derepresses tumor cell migration. S100A4(+)/HIF-1α(−) tumor cells are subsequently recruited to, and migrate along, preexisting vessels in the presence of extracellular VEGF released by S100A4(+)/HIF-1α(+) GBM cells. This in turn results in tumor progression through acceleration of vascularization.

## Supplementary Information

**Additional file 1: Figure S1.** Co-immunoprecipitation of S100A4 and NMIIA in GBM cells. Western blotting (WB) with anti-NMIIA (upper) and anti-S100A4 antibodies (lower) after immunoprecipitation (IP) with the indicated antibodies using KS-1 cell lysates. Input represents 5% of the total cell extract. Normal rabbit IgG was used as a negative control.

**Additional file 2: Figure S2.** GSC properties in GBMs. (A) Left: staining by HE and IHC for Nestin in GBMs. Note the strong Nestin immunoreactivity in non-Ps and Ps perinecrotic lesions. Necrotic areas are indicated by asterisks and partitioned by dotted lines. Insets show the magnified views of the boxed areas. Original magnification, × 100 and × 400 (inset). Scale bar = 200 μm. Right: IHC score for the indicated molecules in non-Ps and Ps perinecrotic and non-necrotic lesions (non-Ps, Ps, and non-nec). The data are presented as means ± SDs. (B) Correlations between IHC scores of Nestin and related molecules in GBM tissues. *ρ*, Spearman’s correlation coefficient; n, number of cases.

**Additional file 3: Figure S3.** Association between knockdown of S100A4 and glioma stem cell properties. (A) Western blot analysis for the indicated proteins of total lysates from KS-1 cells treated with 100 and 200 μM CoCl_2_. (B) Western blot analysis for the indicated proteins of total lysates from S100A4 knockdown KS-1 cells (KS-shS100A4#3 and #5) and control cells (Con). (C) Left: phase-contrast photograms of spheroids following control or S100A4 knockdown in KS-1 cells after 2 weeks of growth. Right: the numbers of spheroids are presented as means ± SDs. (D) Aldefluor analysis of control or S100A4 knockdown KS-1 cells. Cells negative for ALDH1 activity are located in the area to the far left of each plot, and the positive cells are demarcated by the black gate (R1). The percentage of live single-cell population contained in each gate is shown. DEAB, diethylaminobezaldehyde.

## Data Availability

Data and materials will be shared.

## Abbreviations

GBM: Glioblastoma
Ps: Pseudopalisading
VEGF: Vascular endothelial growth factor
GSC: Glioma stem cell
NMIIA: Non-muscle myosin IIA
HIF: Hypoxia-inducible factor
ALDH: Aldehyde dehydrogenase
GFAP: Glial fibrillary acid protein
SMA: Smooth muscle actin
CA9: Carbonic anhydrase 9
CoCl_2_: Cobalt chloride
IP: Immunoprecipitation
MYH9: Myosin heavy chain 9
KD: Knockdown
HE: Hematoxylin and eosin
IHC: Immunohistochemistry
OS: Overall survival
PFS: Progression-free survival
MVD: Microvascular density
