# Supplementary figures and images for: A functional role of S100A4/non-muscle myosin IIA axis for pro-tumorigenic vascular functions in glioblastoma
Source: Cell Commun Signal. 2022 Apr 7;20:46. doi: 10.1186/s12964-022-00848-w (PMC8991692; doi:10.1186/s12964-022-00848-w)

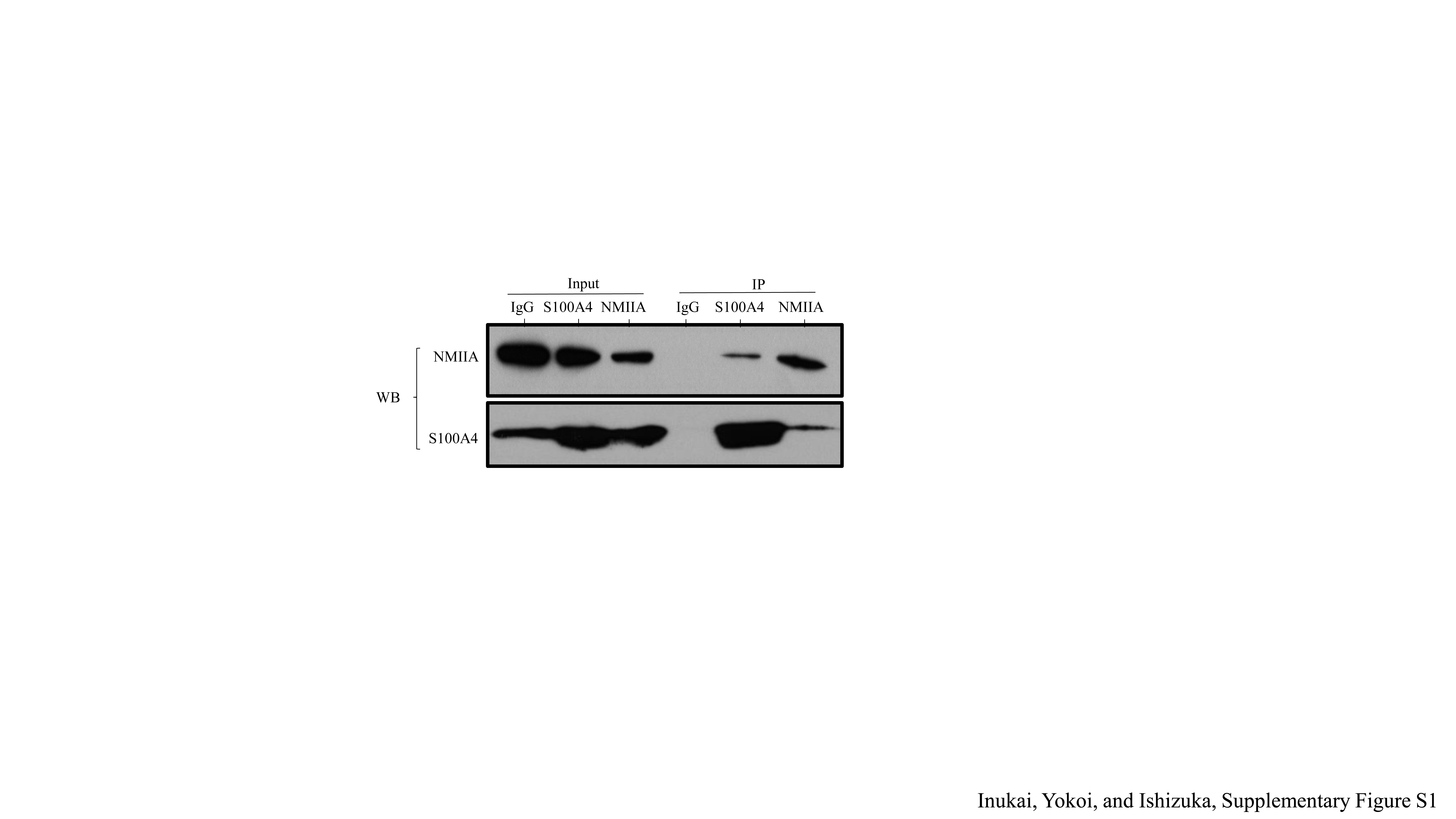

Supplement: Supplementary file 2 — Additional file 1: Figure S1. Co-immunoprecipitation of S100A4 and NMIIA in GBM cells. Western blotting (WB) with anti-NMIIA (upper) and anti-S100A4 antibodies (lower) after immunoprecipitation (IP) with the indicated antibodies using KS-1 cell lysates. Input represents 5% of the total cell extract. Normal rabbit IgG was used as a negative control. [file 12964_2022_848_MOESM2_ESM.tif]

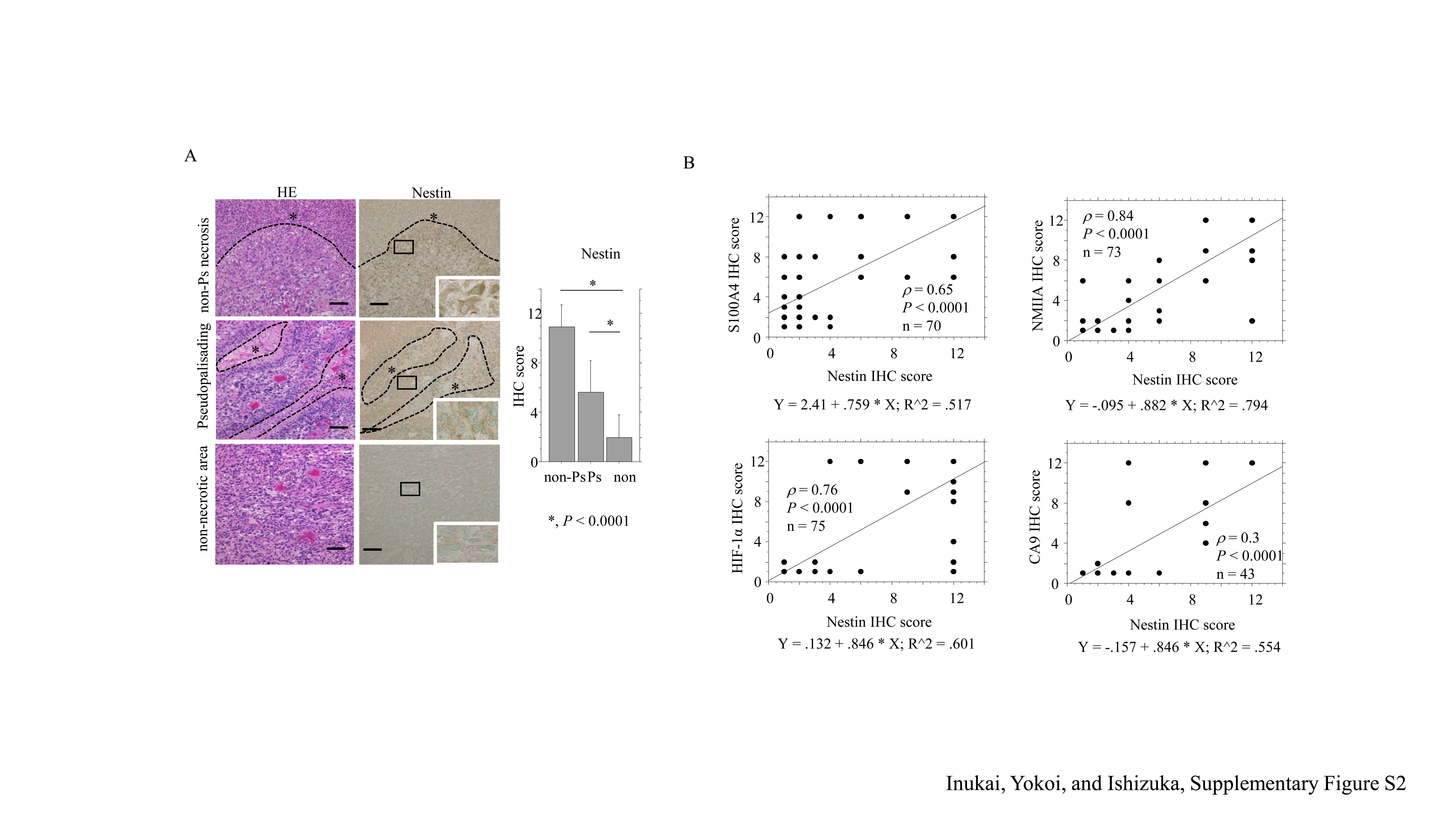

Supplement: Supplementary file 3 — Additional file 2: Figure S2. GSC properties in GBMs. (A) Left: staining by HE and IHC for Nestin in GBMs. Note the strong Nestin immunoreactivity in non-Ps and Ps perinecrotic lesions. Necrotic areas are indicated by asterisks and partitioned by dotted lines. Insets show the magnified views of the boxed areas. Original magnification, × 100 and × 400 (inset). Scale bar = 200 μm. Right: IHC score for the indicated molecules in non-Ps and Ps perinecrotic and non-necrotic lesions (non-Ps, Ps, and non-nec). The data are presented as means ± SDs. (B) Correlations between IHC scores of Nestin and related molecules in GBM tissues. ρ, Spearman’s correlation coefficient; n, number of cases. [file 12964_2022_848_MOESM3_ESM.tif]

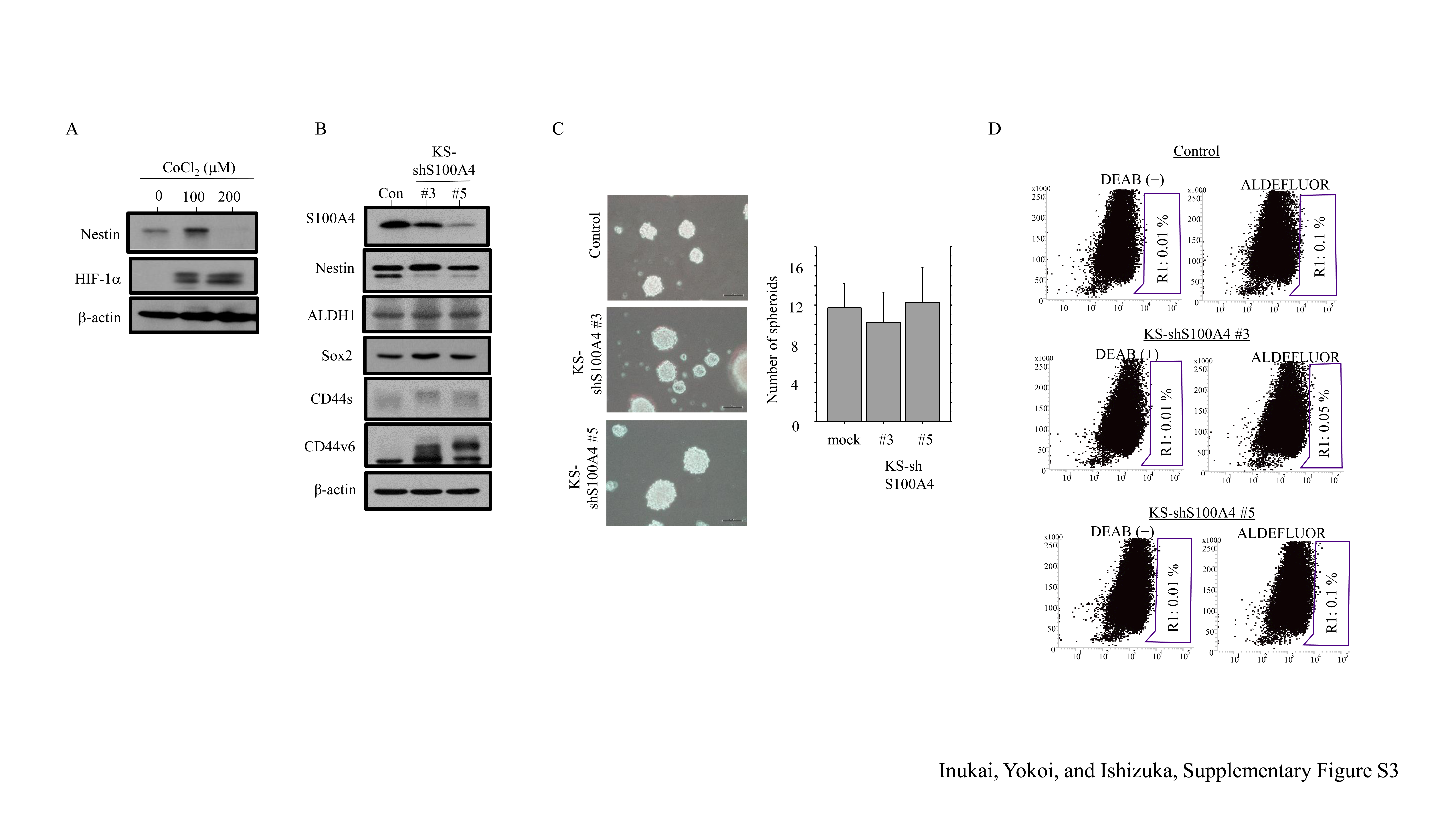

Supplement: Supplementary file 4 — Additional file 3: Figure S3. Association between knockdown of S100A4 and glioma stem cell properties. (A) Western blot analysis for the indicated proteins of total lysates from KS-1 cells treated with 100 and 200 μM CoCl2. (B) Western blot analysis for the indicated proteins of total lysates from S100A4 knockdown KS-1 cells (KS-shS100A4#3 and #5) and control cells (Con). (C) Left: phase-contrast photograms of spheroids following control or S100A4 knockdown in KS-1 cells after 2 weeks of growth. Right: the numbers of spheroids are presented as means ± SDs. (D) Aldefluor analysis of control or S100A4 knockdown KS-1 cells. Cells negative for ALDH1 activity are located in the area to the far left of each plot, and the positive cells are demarcated by the black gate (R1). The percentage of live single-cell population contained in each gate is shown. DEAB, diethylaminobezaldehyde. [file 12964_2022_848_MOESM4_ESM.tif]
